# Detection of circulating natural antibodies to inflammatory cytokines in type-2 diabetes and clinical significance

**DOI:** 10.1186/s12950-017-0171-6

**Published:** 2017-11-06

**Authors:** Weiyi Cai, Cailing Qiu, Hongyu Zhang, Xiangyun Chen, Xuan Zhang, Qingyong Meng, Jun Wei

**Affiliations:** 1 Laboratory for Nursing Science & Institute of Laboratory Medicine, Guangdong Medical University, No.1 Xincheng Road, Dongguan, 523808 China; 2 Dalang Hospital of Dongguan, Dongguan, 523000 China; 3 The Second Hospital, Jilin University, Changchun, 130041 China; 4 Division of Health Research, University of the Highlands & Islands, Centre for Health Science, Perth Road, Inverness, IV2 3JH UK

**Keywords:** Natural antibodies, IgG antibody, Inflammatory cytokines, Type-2 diabetes, ELISA

## Abstract

**Background:**

Inflammatory cytokines have been demonstrated to be involved in developing insulin resistance and type-2 diabetes (T2D). Natural antibodies in the circulation have protective effects on common diseases in humans. The present study was thus designed to test the hypothesis that natural antibodies against inflammatory cytokines could be associated with T2D.

**Methods:**

An enzyme-linked immunosorbent assay (ELISA) was developed in-house to detect plasma IgG against peptide antigens derived from interleukin 1α (IL1α), IL1β, IL6, IL8 and tumor necrosis factor-α (TNF-α) in 200 patients with T2D and 220 control subjects.

**Results:**

Binary regression showed that compared with control subjects, T2D patients had a decreased level of plasma anti-IL6 IgG (adjusted *r* ^2^=0.034, *p*=0.0001), anti-IL8 IgG (adjusted *r* ^2^=0.021, *p*=0.002) and anti-TNF-α IgG (adjusted *r* ^2^=0.017, *p*=0.003). Female patients mainly contributed to decreased levels of anti-IL6 IgG (adjusted *r* ^2^=0.065, *p*=0.0008) and anti-IL8 IgG (adjusted *r* ^2^=0.056, *p*=0.003), while male patients mainly contributed to decreased anti-TNF-α IgG levels (adjusted *r* ^2^=0.024, *p*=0.005). ROC curve analysis revealed a sensitivity of 16.5% against specificity of 95.5% for anti-IL6 IgG assay and a sensitivity of 19.5% against specificity of 95.9% for anti-IL8 IgG assay. Glycated hemoglobin levels measured after 6-month glucose-lowering treatment appeared to be inversely correlated with plasma anti-IL1α IgG (*r*=-0.477, df=17, *p*=0.039) and anti-IL6 IgG (*r*=-0.519, df=17, *p*=0.023) although such correlation failed to survive the Bonferroni correction.

**Conclusions:**

Deficiency of natural IgG against inflammatory cytokines is likely to be a risk factor for T2D development and detection of such antibodies may be useful for personalized treatment of the disease.

**Electronic supplementary material:**

The online version of this article (10.1186/s12950-017-0171-6) contains supplementary material, which is available to authorized users.

## Background

Type-2 diabetes (T2D) is a metabolic disorder that results from interaction between genetic predisposition and environmental components [1]. The prevalence of T2D is rising globally, accounting for 85%–95% of all forms of diabetes in developed countries and a higher percentage in developing countries [2]. China has become a global epicenter of diabetes; a national study from June 2007 through May 2008 demonstrated that the prevalence of diabetes was 9.7% among Chinese adults [3]. Obesity is a major risk factor for T2D and obesity-induced chronic inflammation is likely to play a key role in the pathogenesis of insulin resistance that leads to the development of T2D [4, 5]. Multiple inflammatory inputs contribute to metabolic dysfunction [6]. The World Health Organization (WHO) published an epidemiological report in 2014, indicating that more than 1.9 billion adult people (18 years and older) in the world were overweight, 600 millions of whom were obese (http://www.who.int/mediacentre/factsheets/fs311/en/index.html). It is worth noting that approximately one third of the population is overweight or obese, but only ~5% develops T2D. There must be something in our body to regulate susceptibility to T2D in obese people.

Inflammatory cytokines, such as interleukin 1 (IL1), IL6 and tumor necrosis factor alpha (TNF-α) have been confirmed to be involved in developing insulin resistance [7]. Circulating IL8 was also found to be elevated in patients with T2D and associated with obesity-related parameters [8, 9]. Anti-inflammatory system could reduce susceptibility to insulin resistance and T2D. It has been indicated that the blockage of inflammatory cytokines has T2D-protective characteristics [4, 10]. Monoclonal antibodies targeting inflammatory cytokines have been used to treat coronary artery disease [11], rheumatoid arthritis and other systemic autoimmune diseases [12, 13]. Natural antibodies are defined as immunoglobulins that are spontaneously and constitutively secreted by the B1 type of lymphocytes in the absence of external antigen stimulation or immunization [14, 15]. Natural antibodies play an important role not only in eliminating pathogens invaded but also in maintaining homeostasis of the immune system by prevention of autoimmune and inflammatory reactions [16–19]. Possibly, the content of natural antibodies is in inverse proportion to inflammatory cytokines, and a decrease in natural antibody levels may lead to increased activities of inflammatory cytokines [20–22]. From an immunological point of view, the effects of inflammatory cytokines on our body may depend on circulating levels of their corresponding natural antibodies [15]. Accordingly, the present work was undertaken to examine if circulating levels of natural antibodies against inflammatory cytokines were associated with T2D and the therapeutic effects of glucose-lowering treatment on the disease in a Chinese population.

## Methods

### Subjects

A total of 200 patients aged 48.2±7.2 years, who were diagnosed as having T2D, and 220 healthy controls aged 44.2±8.6 years, were recruited by Dalang Hospital of Dongguan, China for this study. These 200 patients had a disease duration of 1.9±2.4 years on average, ranging from 0 to 9.4 years and they all fulfilled the diagnostic criteria for diabetes published by the WHO, 2006. (http://www.who.int/diabetes/publications/diagnosis_diabetes2006/en/). Those patients who developed clinical complications or suffered from autoimmune disease, malignant disease and mental illness, were excluded from this study. Glycated hemoglobin (HbA1c) levels in blood were taken from clinical records if available, which were measured before glucose-lowering treatment and in 3 and 6 months of post-treatment. Control subjects did not have history of diabetes, autoimmune disease, malignant illness and mental disorders. There were 54 smokers (27.0%) in the patient group and 60 smokers (27.3%) in the control group. All the subjects were of Chinese Han origin, and all gave written informed consent to participate in this study. This work was approved by an Institutional Review Board and conformed to the requirements of the Declaration of Helsinki.

### Antibody testing

Five linear peptide antigens that are respectively derived from IL1α, IL1ß, IL6, IL8 and TNF-α, were designed according to the computational prediction of human leukocyte antigen (HLA) class II epitopes [23, 24], and their amino acid sequences are given in Table 1. An enzyme-linked immunosorbent assay (ELISA) was developed in-house based on a recent publication [25]. Briefly, solid-phase synthetic peptides were dissolved in 67 % acetic acid to obtain a concentration of 5mg/ml as stock solution. Maleimide-activated plates (Thermo Scientific, Shanghai, China) were coated according to the Manufacturer’s instruction. The antigen-coated plates were washed twice with 200μl Wash Buffer that was phosphate-buffered saline (PBS) (P4417, Sigma-Aldrich, Shanghai, China) containing 0.05% Tween-20; 50μl plasma sample diluted 1:200 in Assay Buffer that was PBS containing 0.5% bovine serum albumin (BSA) was then added to each sample well; 50μl Assay Buffer was added to each negative control (NC) well and 50μl positive control (PC) sample was added to each PC well. Following incubation at room temperature for 1.5 hours, the plate was washed three times with 200μl Wash Buffer and 50μl peroxidase-conjugated goat anti-human IgG antibody (ab98567, Abcam, Guangzhou, China) diluted 1:50000 in Assay Buffer was added to each well. After incubation at room temperature for 1 hour, color development was initiated by adding 50μl Stabilized Chromogen (SB02, Life Technologies, Guangzhou, China) and terminated after 20 min by adding 25μl Stop Solution (SS04, Life Technologies). The measurement of optical density (OD) was completed on a microplate reader within 10 min at 450nm with a reference wavelength of 620nm. All the samples were tested in duplicate and the specific binding ratio (SBR) was used to represent the relative levels of plasma IgG antibodies. Calculation of SBR is as follows: SBR= (OD _Sample_ – OD _NC_) / (OD _PC_ – OD _NC_)

**Table 1:** Sequence of peptide antigens used for in-house ELISA

Antigen	Sequence
IL1α	LLFFWETHGTKNYFTSVAHPNLFIATKQDYWVCLAGGP
IL1β	LNCTLRDSQQKSLVMSGPYELKALHLQGQDMEQQVVF
IL6	TCLVKIITGLL EFEVYLEYLQNRFESSEEQARAVQM
IL8	ELRCQCIKTYSKPFHPKFIKELRVIESGPH
TNF-α	LIYSQVLFKGQGCPSTHVLLTHTISRIAVSYQTKVNLLS

To minimize an intra-assay deviation, the ratio of the difference between duplicated OD values of each sample to their sum was used to assess the precision for the in-house ELISA antibody test. If the ratio was found to be >10%, the test of this sample was treated as being invalid and was not used for data analysis.

### Data analysis

The coefficient of variation (CV) was used to represent an inter-assay deviation estimated using pooled plasma, namely quality control (QC) sample, which was randomly collected from >20 healthy subjects and tested on every 96-well plate. Binary regression analysis was applied to examine the differences in circulating IgG levels between T2D patients and control subjects, with adjustment for age in either male or female samples, and for gender and age in combined samples. Pearson correlation analysis was performed to examine the correlation between plasma IgG levels and the duration of T2D or HbA1c levels in blood. Receiver operating characteristic (ROC) curve analysis was applied to estimate the areas under the ROC curve (AUC) with calculation of ELISA sensitivity against a specificity of ≥95% as well as 95% confident interval (CI). Because five antigens were tested in this study, the p-value of 0.01 was considered to be statistically significant based on the Bonferroni correction.

## Results

The CV estimated based on SBR from the QC sample was 16.2% for anti-IL1α IgG assay, 9.5% for anti-IL1β IgG assay, 8.6% for anti-IL6 IgG assay, 13.9% for anti-IL8 IgG assay and 11.7% for anti-TNF-α IgG assay (Table 2), suggesting that the in-house ELISA developed with linear peptide antigens was highly reproducible.

**Table 2:** Inter-assay deviation between ELISA-testing plates

Antigen	Number of plates	Mean ± SD^a^	CV (%)
IL1α	20	1.70±0.28	16.2
IL1β	23	1.12+0.11	9.5
IL6	20	1.38+0.12	8.6
IL8	20	1.41+0.17	11.7
TNF-α	23	1.33+0.19	13.9

^a^ *SD* standard deviation

Binary regression analysis showed that compared with control subjects, T2D patients had a decreased level of circulating IgG against peptide antigens derived from IL6 (adjusted *r* ^2^=0.034, *p*=0.0001), IL8 (adjusted *r* ^2^=0.021, *p*=0.002) and TNF-α (adjusted *r* ^2^=0.017, *p*=0.003). Female patients mainly contributed to decreased levels of anti-IL6 IgG (adjusted *r* ^2^=0.065, *p*=0.0008) and IL8 IgG (adjusted *r* ^2^=0.056, *p*=0.003), while male patients mainly contributed to decreased anti-TNF-α IgG levels (adjusted *r* ^2^=0.024, *p*=0.005). Circulating IgG against peptide antigens derived from IL1α and IL1β did not show a significant change in patients with T2D (Table 3).

**Table 3:** Binary regression analysis of circulating IgG against inflammatory cytokines in T2D

Antigens	Patient (*n*)	Control (*n*)	Adjusted *r* ^2^	*p* ^a^
IL1α				
Male	1.15±0.42 (124)	1.21±0.39 (131)	-0.002	0.228
Female	1.20±0.43 (76)	1.32±0.39 (89)	0.009	0.083
Combined	1.17±0.42 (200)	1.26±0.39 (220)	0.012	0.04
IL1β				
Male	0.70±0.28 (124)	0.77±0.30 (131)	0.019	0.04
Female	0.78±0.31 (76)	0.80±0.33 (89)	-0.01	0.788
Combined	0.73±0.29 (200)	0.78±0.31 (220)	0.011	0.07
IL6				
Male	1.13±0.21 (124)	1.18±0.19 (131)	0.01	0.038
Female	1.11±0.23 (76)	1.24±0.22 (89)	0.065	0.0008
Combined	1.12±0.22 (200)	1.20±0.21 (220)	0.034	0.0001
IL8				
Male	0.97±0.35 (124)	1.02±0.29 (131)	0.003	0.134
Female	0.94±0.33 (76)	1.12±0.35 (89)	0.056	0.003
Combined	0.96±0.34 (200)	1.06±0.32 (220)	0.021	0.002
TNF-α				
Male	0.77±0.40 (124)	0.95±0.60 (131)	0.024	0.005
Female	0.85±0.48 (76)	0.95±0.45 (89)	-0.002	0.226
Combined	0.80±0.43 (200)	0.95±0.54 (220)	0.017	0.003

Data were expressed as mean±SD. ^a^Adjusted for age in male and female samples, and for gender and age in combined samples.

As shown in Table 4, none of plasma IgG antibodies for these five inflammatory cytokines was significantly correlated with HbA1c levels measured either before glucose-lowering treatments or after 3-month treatment. However, HbA1c levels measured after 6-month glucose-lowering treatment appeared to be inversely correlated with anti-I IL1α IgG (*r*=-0.477, df=17, *p*=0.039) and anti-IL6 IgG (*r*=-0.519, df=17, *p*=0.023), although such significant correlations failed to survive the Bonferroni correction (*p*>0.01).

**Table 4:** Correlation between plasma IgG and HbA1c levels in T2D patients before and after glucose-lowering treatment

Antigen	Before treatment		3-month treatment		6-month treatment	
	*r*	*p* ^a^	*r*	*p* ^b^	*r*	*p* ^c^
IL1α	-0.024	0.762	-0.005	0.978	-0.477	0.039
IL1β	-0.011	0.888	-0.030	0.875	-0.278	0.250
IL6	-0.052	0.517	-0.191	0.304	-0.519	0.023
IL8	-0.089	0.266	-0.239	0.196	-0.400	0.090
TNF-α	0.069	0.384	-0.129	0.489	-0.137	0.576

^a^df=157; ^b^df=29; ^c^df=17

**Fig. 1:**
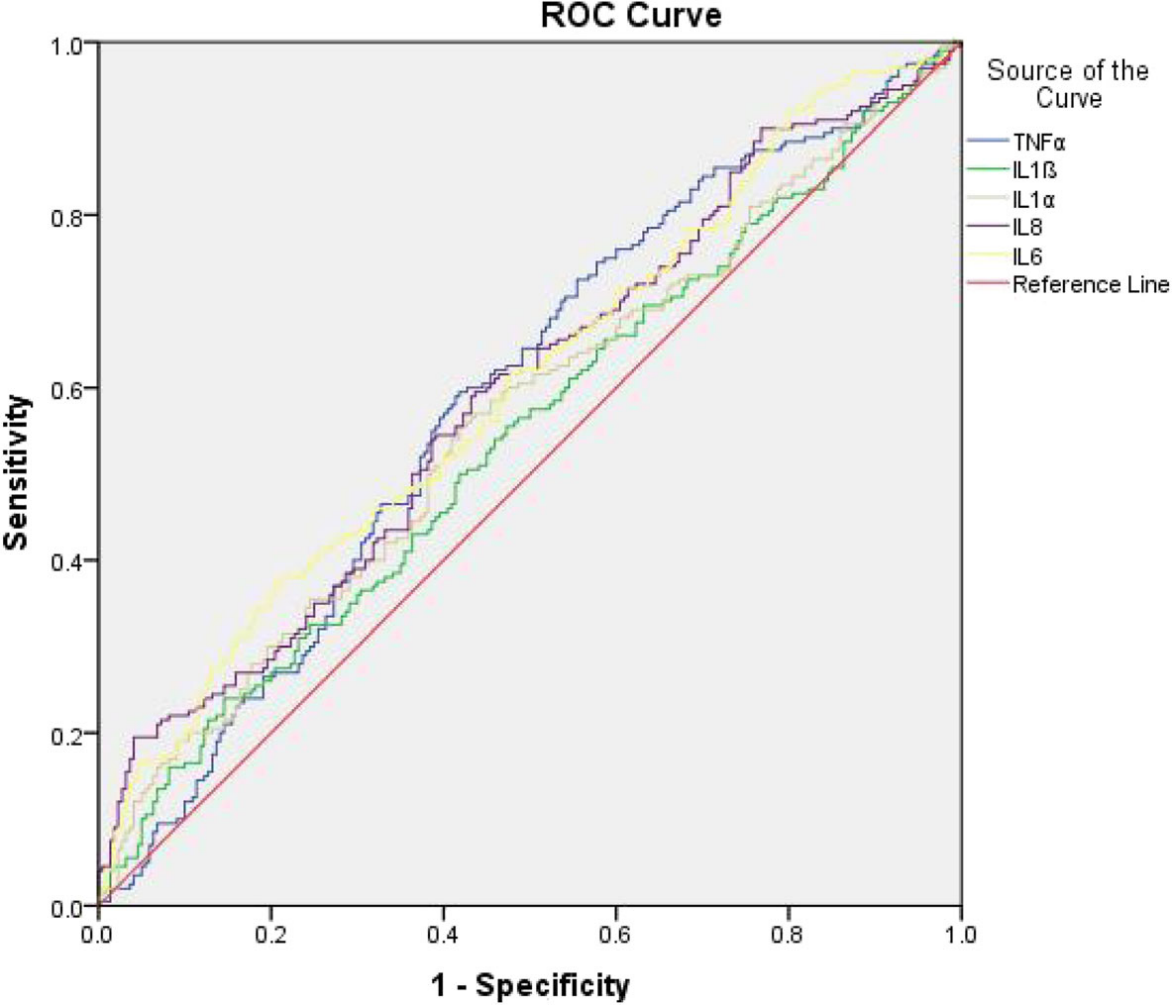
ROC curve analysis of circulating IgG against inflammatory cytokines in T2D. Anti-IL1α IgG had an AUC of 0.564 (95% CI 0.509-0.619) with sensitivity of 13.0% against a specificity of 95.0%; anti-IL1β IgG had an AUC of 0.544 (95% CI 0.489-0.599) with sensitivity of 10.0% against a specificity of 95.0%; anti-IL6 IgG had an AUC of 0.601 ( 95% CI 0.547-0.655) with sensitivity of 16.5% against a specificity of 95.5%; anti-IL8 IgG had an AUC of 0.593 (95%CI 0.538-0.647) with sensitivity of 19.5% against a specificity of 95.9%; anti-TNF-α IgG had an AUC of 0.589 (95%CI 0.534-0.643) with sensitivity of 4.5% against a specificity of 95.0%

**Fig. 2:**
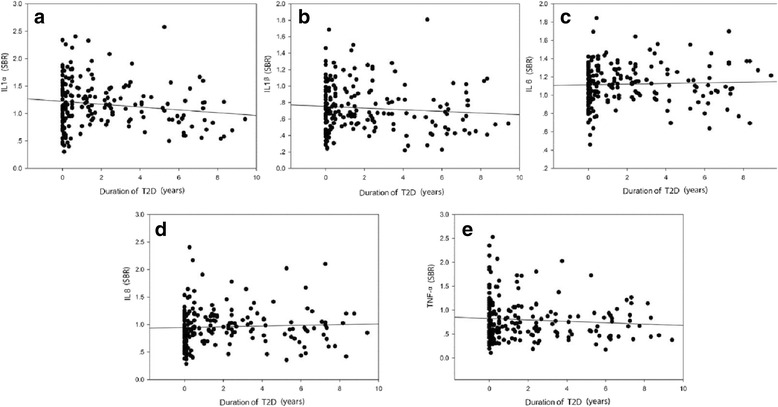
Correlation between the duration of T2D and the levels of circulating IgG against inflammatory cytokines **a**. IL1α IgG : *r* = -0.148, df =199, *p*= 0.037; **b**. IL1β IgG: *r* = -0.085, df = 199, *p*= 0.234; **c**. IL6 IgG: *r* = 0.04, df =199, *p*= 0.576; **d**. IL8 IgG: *r* = 0.047, df = 199, *p*= 0.508; e. TNF-α IgG: *r* = -0.083, df = 199, *p*= 0.242

ROC curve analysis revealed that anti-IL6 IgG assay had an AUC of 0.601 (95% CI 0.55-0.66) with sensitivity of 16.5% against a specificity of 95.5%, and anti-IL8 IgG assay had an AUC of 0.593 (95% CI 0.54-0.65) with sensitivity of 19.5% against a specificity of 95.9%; all other three IgG tests had a sensitivity of less than 15% against the specificity of 95% (Fig. 1). Pearson correlation analysis failed to show a significant correlation between the duration of T2D and the levels of circulating IgG against inflammatory cytokines (Fig. 2).

## Discussion

Natural antibodies are present in both animals and humans; they are thought to comprise the bulk of resting IgM, along with portions of isotype-switched IgA and IgG [26, 27]. Natural antibodies also tend to be autoreactive and perform a second beneficial function in housekeeping and homeostatic activity for elimination of dying cells and noxious molecular species [16, 28]. It is believed that natural antibodies are related to the most common, distressing and burdensome diseases, majority of which is associated with aging [15]. In this study, we found that deficiency of natural antibodies against IL6, IL8 and TNF-α was associated with T2D (Table 3) although their levels in the circulation may not be correlated with the duration of T2D (Fig. 2). ROC curve analysis revealed a sensitivity of >15% against the specificity of >95% for both the anti-IL6 assay and the anti-IL8 IgG assay. The sensitivity may represent a clinical subgroup that has undergone an inflammatory process in patients with T2D. Interestingly, HbA1c levels in blood are inversely correlated with plasma anti-IL1α and anti-IL6 IgG levels (Table 4). These observations raise the possibility that natural antibodies against inflammatory cytokines may be useful biomarkers for the development of personalized treatment of T2D on the one hand, and on the other hand, they can also serve as a key component in the body to bridge the gap between T2D and obesity, and counteract the onset of obesity-related diseases.

Gender differences in insulin resistance and T2D have been observed in several studies [29–32]. Women with T2D generally have poor glycemic control and are less likely to reach the goals for HbA1c, as compared with men [30]. Diversities in biology, culture, lifestyle, environment and socioeconomic status impact the differences in risk, pathophysiology and complications of T2D between male and female patients [32]. In this study, we found that there was a gender difference in circulating IgG antibodies against inflammatory cytokines (Table 3); decreased levels of anti-IL6 and anti-IL8 IgG antibodies were more likely to occur in female than male patients, whereas deficiency of anti-TNF-α IgG was more likely to be observed in male than female patients. The gender differences in natural IgG antibodies against inflammatory cytokines provide a clue to the insight into the pathological mechanism behind T2D development in humans. To our knowledge, this is the first report on a decrease in circulating IgG antibodies against inflammatory cytokines. However, this is a piece of preliminary work and the initial finding needs further replication in a large sample size and also in subpopulations.

## Conclusions

Deficiency of natural IgG against inflammatory cytokines is likely to be a risk factor for T2D development and detection of such antibodies may be useful for personalized treatment of the disease.

## Additional file

Additional file 1: Supplementary information. (XLSX 195 kb)

## Abbreviations

AUC: Areas under the ROC curve
BSA: Bovine serum albumin
CI: Confident interval
CV: Coefficients of Variation
ELISA: Enzyme-linked immunosorbent assay
HbA1c: Glycated hemoglobin
HLA: Human leukocyte antigen
IgA: Immunoglobulin A
IgG: Immunoglobulin G
IgM: Immunoglobulin M
IL1α: Interleukin 1α
IL1β: Interleukin 1β,
IL6: Interleukin 16
IL8: Interleukin 8
NC: Negative control
OD: Optical density
PBS: Phosphate -buffered saline
PC: Positive control
QC: Quality control
ROC: Receiver operating characteristic curve
SBR: Specific binding ratio
T2D: Type-2 diabetes
TNF-α: Tumor necrosis factor-α
WHO: World Health Organization
